# Robotic Full-Thickness Resection of a Type IV Gastric Gastrointestinal Stromal Tumor (GIST) Through Organoaxial Gastric Rotation: Technical Description With Video and Operative Management Insights

**DOI:** 10.7759/cureus.95682

**Published:** 2025-10-29

**Authors:** Irene Fiume, Filippo Petrelli, Alberto Patriti

**Affiliations:** 1 Department of General and Oncologic Surgery, AST Pesaro-Urbino, Pesaro, ITA

**Keywords:** indocyanine green fluorescence, intracorporeal suturing, organoaxial rotation, robotic resection, type iv gastric gist

## Abstract

Gastrointestinal stromal tumors (GISTs) of the stomach are increasingly managed using minimally invasive approaches. Tumors located on the posterior gastric wall (type IV) pose significant challenges due to limited exposure and difficult angulation. Robotic surgery may offer technical advantages in such anatomically complex cases.

We describe a case of robotic full-thickness resection of a posterior wall gastric GIST using organoaxial gastric rotation to optimize exposure. In type IV gastric GIST, organoaxial gastric rotation plays a pivotal role, facilitating the exposure of posterior lesions without anterior gastrotomy or excessive mobilization. Robotic surgery ensures stable operative field exposure and precise dissection and suturing in anatomically challenging regions.

## Introduction

Gastrointestinal stromal tumors (GISTs) represent the most common mesenchymal neoplasms of the gastrointestinal tract, with the stomach being the most frequent site of origin [1].

According to population-based cohort studies, the reported annual incidence of GISTs ranges from 4 to 22 cases per million inhabitants, with most studies converging on an incidence between 10 and 15 cases per million. This variability largely reflects differences in diagnostic awareness and methodology, rather than true epidemiological variation.

Regarding tumor localization, approximately 55%-60% of GISTs arise in the stomach, followed by the small intestine (≈30%), while colorectal, esophageal, and other sites are much less frequent [1].

Surgical resection remains the cornerstone of treatment for non-metastatic gastric GISTs, to achieve R0 resection while preserving gastric function [2].

Various minimally invasive techniques, including laparoscopic, robotic, and hybrid surgical-endoscopic approaches, have been described for the treatment of gastric GISTs. Tumor location significantly influences surgical strategy and technical complexity. Mazer et al. [3] emphasized tumor size and anatomical site as key determinants of the appropriate resection type, including wedge, disk, transgastric, or anatomic resections. To support tailored decision-making, Privette et al. [4] proposed a classification system based on tumor location: type I (fundus/greater curvature), type II (antrum), and type III (lesser curvature/gastroesophageal junction). Al-Thani et al. [5] later introduced type IV, referring to tumors on the posterior gastric wall, which are technically challenging due to limited exposure and restricted angulation.

Robotic surgery offers several advantages in this context, including tremor filtration, three-dimensional visualization, articulated instruments, and superior ergonomics, features that are especially valuable in type IV tumors. Prior reports by Arseneaux et al. [6] and Ceccarelli et al. [7] have demonstrated successful robotic resections of GISTs in difficult locations using full-thickness, suture-based techniques.

Here, we describe our technique and provide an accompanying video that introduces the clinical case and illustrates the entire workflow. The video presents the preoperative findings, including endoscopic and contrast-enhanced CT images, confirming the presence of a posterior wall gastric GIST. It then details each intraoperative phase, highlighting the key steps of robotic setup, organoaxial gastric rotation, full-thickness resection, and two-layer intracorporeal closure. The final segment of the video demonstrates intraoperative testing of the suture line with methylene blue and indocyanine green (ICG) fluorescence to confirm integrity and perfusion.

## Technical report

Clinical presentation and operative management

A 62-year-old male patient presented with persistent dyspeptic symptoms characterized by postprandial fullness, epigastric discomfort, and intermittent nausea lasting for approximately eight months. The symptoms were not related to specific foods, showed no improvement after empirical proton pump inhibitor therapy, and were occasionally accompanied by mild bloating. There was no history of vomiting, gastrointestinal bleeding, or weight loss.

His past medical history was notable for chronic demyelinating sensorimotor polyneuropathy, for which he was a carrier of a neurostimulator device, as well as monoclonal gammopathy, paroxysmal atrial fibrillation, and well-controlled arterial hypertension under medical therapy. The patient denied any prior abdominal surgery or family history of gastrointestinal malignancy.

On physical examination, the patient was afebrile, hemodynamically stable, with a soft and non-tender abdomen, no palpable masses, and no hepatosplenomegaly. Bowel sounds were normal.

Given the persistence of symptoms despite medical therapy, he was referred by his primary care physician for gastroenterological evaluation. Before endoscopy, an abdominal ultrasound was performed, which revealed no liver lesions or biliary tract abnormalities but showed a mildly thickened gastric wall in the body region, prompting further endoscopic investigation.

Upper gastrointestinal endoscopy showed a regular esophagus with two small linear erosions in the distal third and a continent squamocolumnar junction. The stomach was normodistensible with normal mucosa throughout; however, in the gastric body along the greater curvature, approximately 5 cm from the cardia, a subepithelial lesion of about 6 cm in diameter with an ulcerated apex was observed. The lesion appeared well-circumscribed, with an overlying mucosa showing a central ulceration and fibrin deposition. Multiple biopsies were obtained from the ulcerated area.

**Figure 1:**
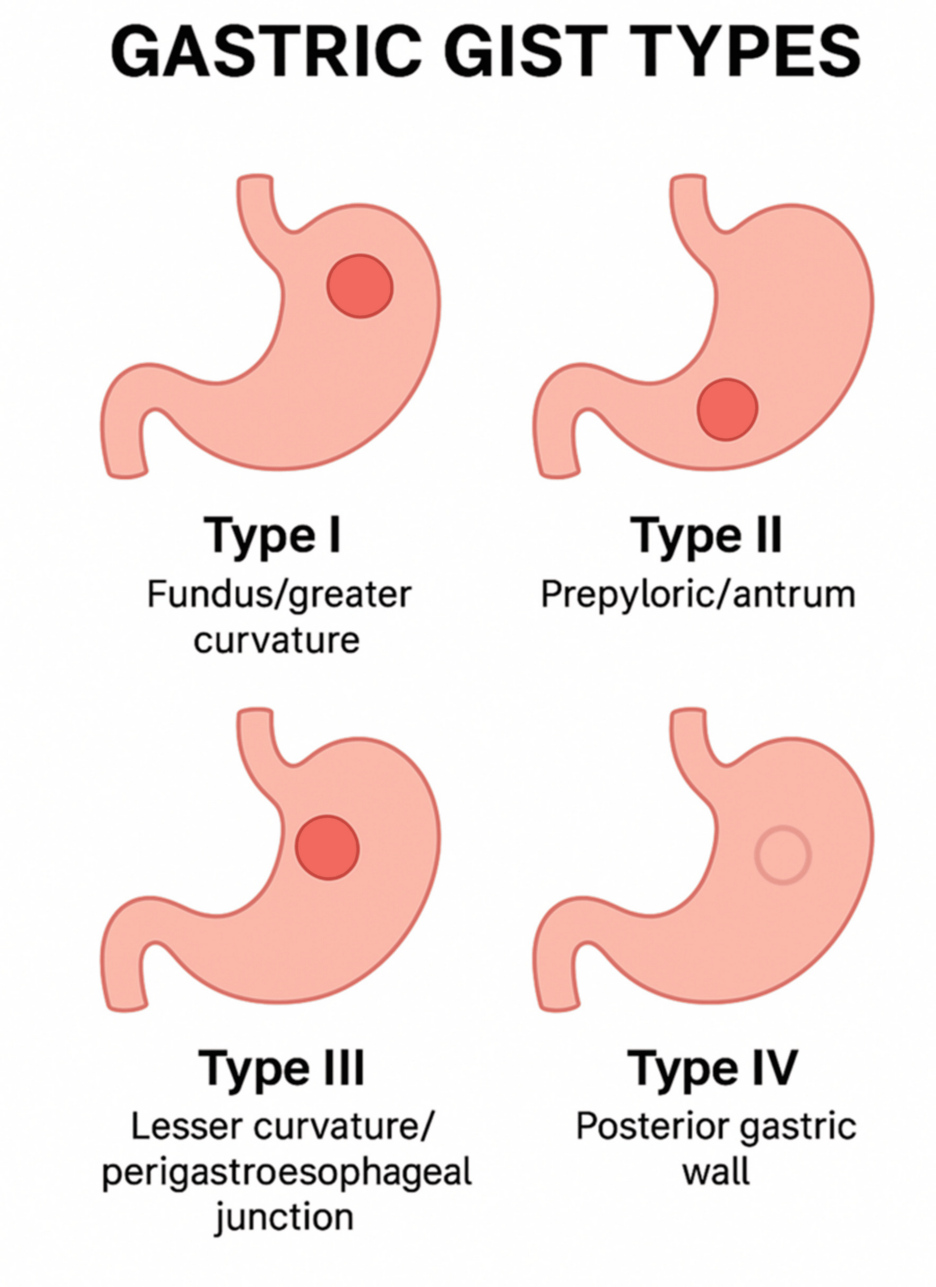
Schematic representation of gastric GIST types according to tumor location and surgical complexity. Type I: fundus/greater curvature; type II: prepyloric/antrum; type III: lesser curvature/perigastroesophageal junction; type IV: posterior gastric wall. Image credit: Original illustration created by the authors, conceptually adapted from the classifications by Privette et al. [4] and Al-Thani et al. [5]. GIST, gastrointestinal stromal tumor

Endoscopic ultrasound confirmed a 5 cm hypoechoic, homogeneous mass arising from the muscularis propria. A contrast-enhanced CT scan excluded metastatic disease and confirmed the presence of a mass on the posterior wall of the stomach near the gastroesophageal junction. The lesion was classified as a type IV gastric GIST, located in a technically challenging position for standard laparoscopic resection (Figure 1).

Technique

**Figure 2:**
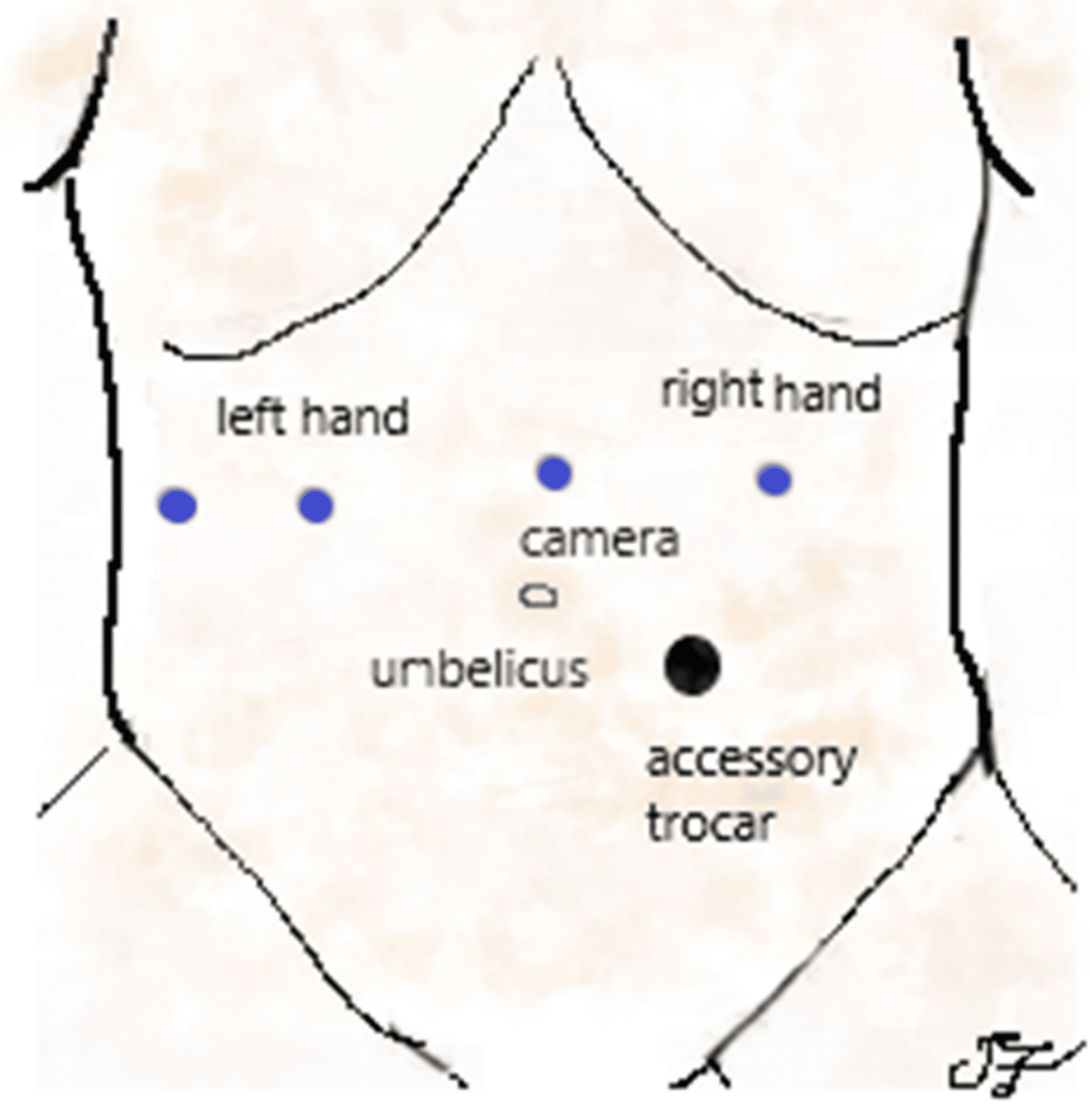
Robotic trocar configuration for gastric GIST resection. Image credit: All authors. GIST, gastrointestinal stromal tumor

The patient was placed in the supine position with reverse Trendelenburg and legs apart. The Da Vinci Xi robotic system was docked from the patient’s right side. A five-port configuration was used. All robotic trocars were aligned along a supraumbilical horizontal line. The camera port was placed in the midline; two robotic trocars were positioned laterally in the right and left upper quadrants, serving as the surgeon’s right- and left-hand instruments. A fourth robotic trocar was inserted in the right flank to elevate the left hepatic lobe. An additional 12 mm assistant trocar was placed infraumbilically, between the right-hand instrument port and the camera port. This port is usually used for suction, suture passage, retraction, and Endobag insertion (Figure 2). At the end of the procedure, the incision at this site was slightly enlarged for specimen extraction.

During induction of anesthesia, a nasogastric tube was inserted by the anesthesiology team to decompress the stomach and facilitate surgical exposure.

The dissection began by mobilizing the greater curvature of the stomach. The gastrosplenic ligament, containing the short gastric vessels, was divided using a robotic Vessel Sealer instrument, which allows simultaneous sealing and cutting of vascular structures, ensuring effective hemostasis. Organoaxial rotation was then performed to expose the posterior wall and bring the tumor into view. A full-thickness resection was carried out using monopolar robotic scissors, without stapler use (Table 1).

**Table 1:** Location of gastric GISTs. Level of complexity refers to visualization, access difficulty, risk of complications (stenosis, deformity), and technical demands of resection. GIST, gastrointestinal stromal tumor

Type	Location	Level of complexity	Usual minimally invasive management
I	Fundus/greater curvature	Low - most favorable location: easy exposure and safe stapled resection margin	Laparoscopic or robotic stapled wedge resection
II	Prepyloric/antrum	Intermediate - technically more demanding due to proximity to the pylorus and risk of postoperative gastric outlet narrowing	Laparoscopic or robotic wedge resection; in selected cases, distal gastrectomy may be required
III	Lesser curvature/perigastroesophageal junction	High - “unfavorable” site; increased risk of luminal deformity and anastomotic tension; included among “difficult cases” in minimally invasive series	Combined laparoscopic-endoscopic (transgastric) resection or advanced robotic approach
IV	Posterior gastric wall	High - site requiring complex exposure; included among “difficult cases” in minimally invasive series	Dedicated robotic approach with organoaxial gastric rotation, or combined laparoscopic/endoscopic transgastric resection

The gastric defect was closed intracorporeally in two layers: an inner running suture for the mucosa and an outer seromuscular reinforcing layer. The stomach was maintained in the rotated position throughout resection and closure to ensure optimal exposure. Leak testing was performed with methylene blue, and vascular perfusion at the suture line is assessed using ICG fluorescence (Video 1).

**Video 1:** Robotic full-thickness resection of type IV gastric GIST using organoaxial rotation. This video highlights the impact of tumor location on surgical planning in gastric GISTs, focusing on type IV lesions of the posterior gastric wall. We detail the technical strategy adopted, including robotic full-thickness resection, organoaxial rotation for exposure, and intracorporeal suturing, with emphasis on the utility of advanced minimally invasive techniques in anatomically challenging settings. GIST, gastrointestinal stromal tumor

Postoperative course

The operative time was 120 minutes. The immediate postoperative course was managed in the local intensive care unit to allow close monitoring and controlled awakening before transfer to the surgical ward. The nasogastric tube was removed on postoperative day one. An upper gastrointestinal contrast study on postoperative day 2 showed no leak and preserved gastric continuity. The patient resumed oral intake and was discharged on postoperative day 2. Histological examination confirmed a gastric GIST with negative margins (R0 resection), characterized by an epithelioid morphology. Immunohistochemical analysis was positive for DOG1 and CD34, with weak or absent KIT (CD117) expression.

Video 1 demonstrates the complete case sequence, including preoperative endoscopic and CT imaging, robotic setup, organoaxial gastric rotation for posterior wall exposure, full-thickness resection, and two-layer intracorporeal closure. The video also includes intraoperative methylene blue and ICG testing of the suture line, followed by postoperative imaging confirming intact closure and normal gastric transit.

## Discussion

The management of gastric GISTs has evolved considerably with the introduction of minimally invasive approaches. Laparoscopic and robotic resections have both demonstrated comparable oncologic outcomes for small and medium-sized gastric GISTs, with lower blood loss, shorter hospital stays, and faster recovery compared to open surgery [8]. However, tumor location remains a major determinant of technical feasibility for gastric GIST resection (Table 1) [3-5]. Posterior wall or gastroesophageal junction lesions (type IV, as defined by Al-Thani et al.) are particularly challenging because of restricted exposure and difficult angulation for stapling or suturing [5]. In such scenarios, robotic technology offers key advantages (enhanced three-dimensional visualization, tremor filtration, and superior ergonomics) that facilitate precise dissection and intracorporeal reconstruction, as emphasized by Arseneaux et al. and Ceccarelli et al. [6,7].

Our case demonstrates how organoaxial gastric rotation, performed robotically, optimizes posterior wall exposure without the need for anterior gastrotomy or extensive gastric mobilization. This maneuver, previously described in hybrid laparoscopic-endoscopic techniques by Maker et al. [9], becomes safer and more reproducible with robotic articulation. The improved dexterity of the robotic platform allows for meticulous full-thickness resection and secure two-layer closure while minimizing manipulation of adjacent structures. This approach aligns with the current trends toward function-preserving surgery, which aims to achieve complete resection (R0) with maximal organ preservation. Recent evidence has reinforced the role of robotic surgery in achieving these goals. Lwin et al. [10] described a series of robotic function-preserving gastric GIST resections that maintained oncologic safety while reducing deformity, promoting physiological gastric emptying, and improving postoperative quality of life. Similarly, Solaini et al. [8] conducted a large multicenter cohort study comparing open, laparoscopic, and robotic resections for gastric GISTs. These authors demonstrated that robotic surgery, despite longer operative times, achieved equivalent oncologic results to open and laparoscopic approaches, with significantly reduced blood loss and shorter hospital stays. Ceccarelli et al. [7] conducted a multi-institutional analysis of a minimally invasive series of gastric resection GISTs performed, which evidenced the advantages of the robotic approach, especially in anatomically unfavorable sites.

Intraoperative leak testing and vascular perfusion assessment are critical in ensuring safe closure after full-thickness resection. In our case, methylene blue testing and ICG fluorescence were used to verify suture integrity and perfusion.

Similar to bariatric surgery and, in particular, sleeve gastrectomy, fluorescence angiography with ICG allows a real-time assessment and gives a direct image of tissue perfusion and vascularization after gastric resection and suturing [11,12]. Although methylene blue leak tests may show variable sensitivity, combining mechanical testing with perfusion imaging may allow immediate identification of potential weak points, allowing for immediate intraoperative correction if needed and therefore enhancing safety in full-thickness gastric resections.

Our postoperative course was uneventful, with early oral intake and radiologic confirmation of intact gastric continuity on oral contrast study. Rapid recovery, absence of leakage on oral contrast study, and early resumption of diet are all findings that align with other robotic series that report low morbidity and favorable short-term outcomes in challenging gastric GISTs [4-7]. However, the current evidence base remains limited to small case series and retrospective analyses, with few prospective or comparative studies. Robotic surgery offers superior dexterity and precision but is associated with higher costs and longer operative times compared with laparoscopy [8,11,13]. Prospective trials and cost-effectiveness studies are necessary to define the true clinical benefits of robotic approaches, particularly for tumors in technically demanding locations.

## Conclusions

Robotic full-thickness resection with organoaxial gastric rotation provides a safe, effective, and function-preserving strategy for Type IV gastric GISTs. This approach combines the oncologic adequacy of traditional resections with the precision and safety of advanced minimally invasive surgery, reducing the need for extensive mobilization. Tailoring the surgical strategy to tumor location remains crucial to achieving optimal oncologic and functional outcomes. The addition of intraoperative perfusion and leak assessment further enhances procedural safety and may contribute to improved postoperative recovery.
